# Fecal Transplants: What Is Being Transferred?

**DOI:** 10.1371/journal.pbio.1002503

**Published:** 2016-07-12

**Authors:** Diana P. Bojanova, Seth R. Bordenstein

**Affiliations:** 1 Department of Biological Sciences, Vanderbilt University, Nashville, Tennessee, United States of America; 2 Department of Pathology, Microbiology, and Immunology, Vanderbilt University, Nashville, Tennessee, United States of America

## Abstract

Fecal transplants are increasingly utilized for treatment of recurrent infections (i.e., *Clostridium difficile*) in the human gut and as a general research tool for gain-of-function experiments (i.e., gavage of fecal pellets) in animal models. Changes observed in the recipient's biology are routinely attributed to bacterial cells in the donor feces (~10^11^ per gram of human wet stool). Here, we examine the literature and summarize findings on the composition of fecal matter in order to raise cautiously the profile of its multipart nature. In addition to viable bacteria, which may make up a small fraction of total fecal matter, other components in unprocessed human feces include colonocytes (~10^7^ per gram of wet stool), archaea (~10^8^ per gram of wet stool), viruses (~10^8^ per gram of wet stool), fungi (~10^6^ per gram of wet stool), protists, and metabolites. Thus, while speculative at this point and contingent on the transplant procedure and study system, nonbacterial matter could contribute to changes in the recipient's biology. There is a cautious need for continued reductionism to separate out the effects and interactions of each component.

## Abstract 2

Fecal transfers are increasingly common in animal models and humans. This essay notes that bacteria may not be the only player in donor feces that can affect the recipient's biology.

## Introduction

**Fig 1:**
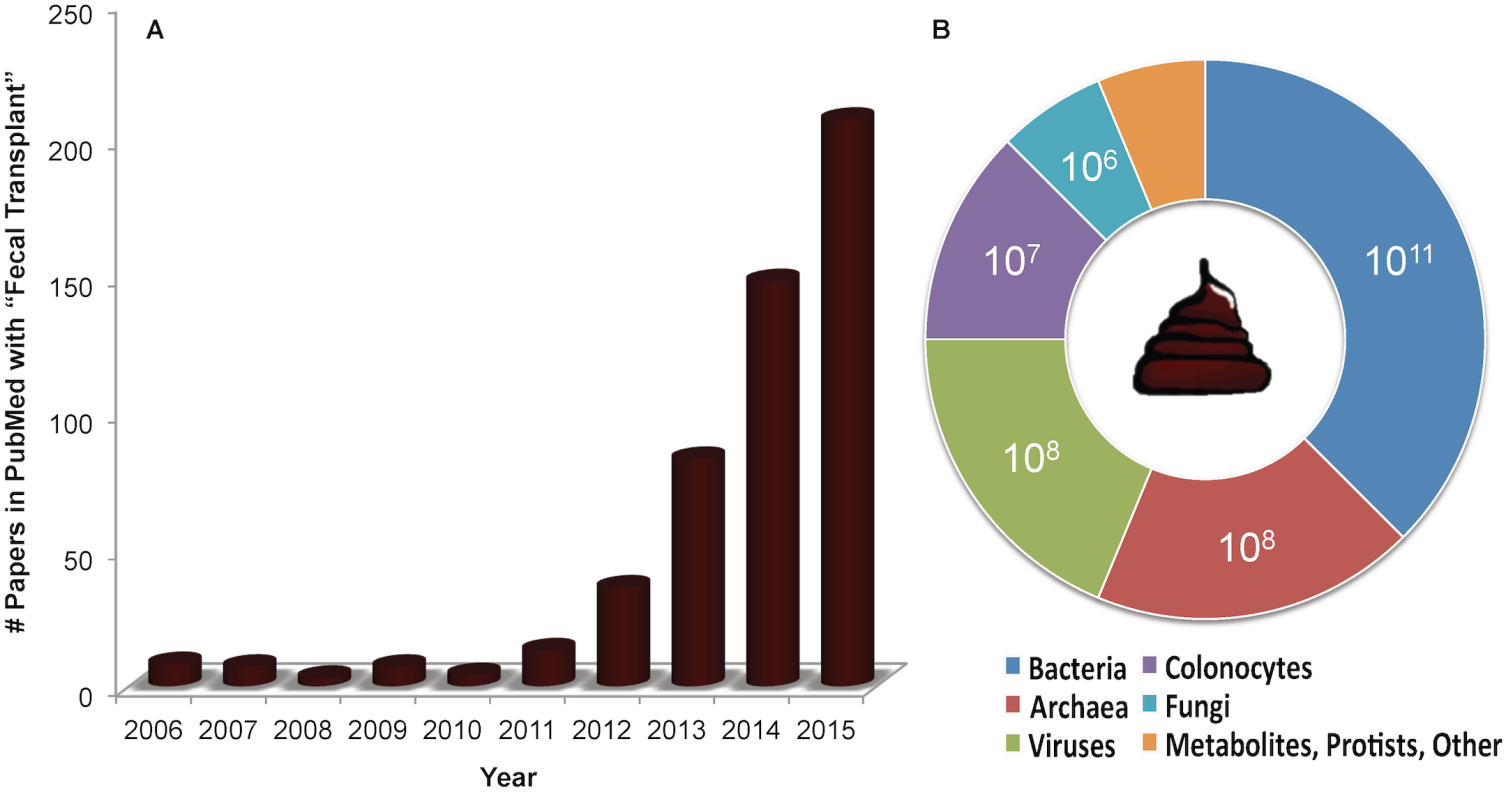
The growth of fecal transplants as reflected in references in PubMed and the estimated composition of human feces. The charts show (A) the rapid rise in publications on fecal transplants in the National Library of Medicine's search service (PubMed), particularly between 2012 and 2015, and (B) the estimated upper concentration of the biological entity per gram of unprocessed human feces, as cited in the text. Estimates do not necessarily reflect the viable number of the biological entity, and the concentration of the archaea is estimated from a methanogen breath test that is not solely based on the presence of archaea. Concentrations of metabolites, protists, and other entities were not identified.

A fecal transplant—the transfer of stool or portions of stool from one organism into the gastrointestinal tract of another—is rapidly gaining attention as a treatment for human gut infections and as a tool for functional "knock-in" studies of the microbiota in animal models. In humans, the procedure is referred to as fecal microbiota transplantation because the microbial components are typically enriched, and in animal models, the transfer of unprocessed stool is commonly achieved by feeding or oral gavage of fecal matter. For the purposes of this essay, we will use the catch-all phrase of “fecal transplants” to refer to all types of procedures. Fig 1 shows the very recent growth of the term in PubMed references involving both human and model system studies.

Several analyses report clinical resolution of *Clostridium difficile* infection (CDI) [1–5], though the long-term effects of the transplants are unknown [6]. Preliminary results also demonstrate positive outcomes for insulin sensitivity [7], multiple sclerosis [8], and Crohn’s disease [9]. The presumptive element connecting these conditions is the gut’s bacterial community, and thus the treatment’s success enthusiastically revolves around intestinal bacteria that are assumed mostly viable in feces. There are a few studies in mice and humans that validate the positive effects of cultured bacteria on CDI [10,11] and mucosal barrier function [12]. Additionally, the microbial portion of human stool can be highly enriched from other fecal material through microfiltration [13,14], spore fractionation [15], and density gradients [16].

Here, we tentatively emphasize that viable bacteria may not be the only player in donor feces that affect the recipient's biology, a fact that is well appreciated by experts. Viruses, archaea, fungi, animal colonocytes, protists, and a number of metabolites that commensal bacteria make or are dependent upon can potentially occur in unprocessed feces. Here, we accentuate the patterns seen in fecal composition analyses and various experiments that illuminate functional effects of individual components of fecal matter. We also highlight important and tractable questions for which further reductionism could help deconstruct the benefit of constituent parts of fecal matter.

## Fecal Composition

Human fecal composition has not been intensively studied. The studies that have examined composition are mostly from the 1970s and 1980s and report varying results, perhaps because of variation in diet and health. On average, adult fecal matter is estimated to be 75% water and 25% solid matter [17]. The vast majority of solid matter is organic material, whose makeup consists of 25%–54% microbial cells (with a slight portion likely consisting of viruses) that may be alive or dead [18]. As microbial counts were based on light microscopy and a modification of the Gram stain, the microbial cells were presumed to be mostly bacteria [18], but quality evidence is lacking. Several other components are found in significant concentration, including archaea, fungi, and microbial eukaryotes. One particular methanoarchaeon species, *Methanobrevibacter smithii*, was detected in 95.7% of patients spanning infants, adults, and the elderly [19], and it can comprise up to 10% of all fecal anaerobes [20]. Viable colonocytes are also readily isolated from newborn and adult feces [21–23]. No analysis of their potential contribution to the success of fecal transplants has been reported. Independent validations of these estimates are needed, particularly measurements that consider all of the entities at once.

While transplants can be highly effective treatment in certain cases, concerns remain about the hypothetical co-transfer of pathogenic microbes [24]. Contamination by environmental microbes is also a risk during the collection, storage, and handling of donor stool, as seen in the early periods of blood storage for transfusions [25,26]. To standardize laboratory protocols and enhance stability of fecal matter, one option is to use frozen donor material from rigorously screened volunteers. Several studies compared the efficacy of frozen versus fresh stool on recurrent or refractory CDI and reported little to no difference [14,27,28]. Extensive longitudinal screening of stool donors is essential to track the long-term success of treatment, and further metagenomic studies of the transferred fecal material and transfer proficiency to recipients are warranted.

### Bacteria

It is well established that the gut contains the highest density of microbes in the human body, with the bacteria-to-human cell ratio recently estimated to be 1.3:1 [29]. In feces, bacteria constitute 25%–54% of solid matter [18] and thus between 6.3% and 13.5% of total fecal matter. Averaged estimates from 14 studies yield a mean bacterial concentration of nearly 10^11^ bacteria per gram of wet stool [29]. Yet, a clear distinction was shown in a study between the viable (49%), injured (19%), and dead (32%) bacterial cells collected from fresh fecal samples under anaerobic conditions [30]. These statistics indicate that only 3.0%–6.6% of total fecal matter may be composed of viable bacteria. The percentage could conceivably be even lower if samples are handled in aerobic conditions for lengthy amounts of time, although frequent aerobic preparation of fecal material has resulted in high cure rates. Furthermore, and as previously noted, transplants with frozen fecal samples that may have reduced viable bacteria can lead to an almost identical resolution of CDI to transplants with fresh samples [27,31]. It should be noted, however, that even bacterial DNA or dead cells might retain some immunostimulatory functions, as colitis symptoms in a dextrose sodium sulfate–induced mouse model were strikingly alleviated by introduction of probiotic DNA and unviable irradiated bacterial cells [32,33].

Other studies suggest that interactions between the host genotype and microbiota can potentially affect transplant outcomes. Across a collection of studies, human fecal donations from related donors showed slightly higher resolution in CDI cases (93%) compared to unrelated donors (84%) [34]. This observation is notable in light of the recent finding that human genetic variation is significantly correlated with variation in bacterial community composition [35,36]. However, a recent meta-analysis demonstrated no significant difference in efficacy between related and unrelated donors [37]. Furthermore, a placebo-controlled trial resulted in the successful treatment of seven of nine people who received a transplant from a single, unrelated, donor [38]. Thus, the evidence to date suggests that relatedness either has little or no effect on treating CDI.

To demonstrate that bacteria directly contribute to disease resolution, several research groups have tested whether enriched bacterial portions of fecal material can be effective in treating CDI in mice and humans. Use of a six-species cocktail therapy suppressed recurrent CDI in 92% of mice [10] when approximately 10^10^ cells per bacterial species were gavaged into recipients. In another mouse study, 10^8^ colony-forming units of a single bacterium isolate, *Lachnospiraceae* D4, caused over a 10-fold reduction in the number of *C*. *difficile* colony-forming units per gram of cecal contents [39]. A cocktail of nontoxigenic *C*. *difficile* spores was also successfully used in suppressing CDI recurrence in a human trial [40]. At 26 weeks of treatment, only 0%–5% of patients from various treatment groups had toxigenic *C*. *difficile* remaining in feces. These studies indicate that cultured bacteria can, in certain cases, be effective contributors to CDI disease resolution.

### Viruses

Viruses from eukaryotes, bacteria, and archaea are less studied components of the gut microbiota than bacteria. From five fecal samples, count estimates indicate that the viral abundance ranges from 10^8^ to 10^9^ viruses per gram of feces (wet weight), and the average virus-to-bacterium ratio is 0.13 [41]. These estimates are comparably low to those reported in other environments where the virus-to-microbial cell ratios range from 1.4 to 160 [42], which supports the emerging view that viruses exhibit a more temperate lifestyle in the gut [43,44]. Additionally, a recent metagenomic study demonstrated that numerous temperate phages are transferred during fecal transplants [24]. Prophages often assist in controlling invading pathogens, modulating community structure, and maintaining gut homeostasis [44]. The dominance of temperate viruses is, however, typical of healthy control feces, as patients suffering from bowel diseases can have increased amounts of virulent phages [45]. One of the most abundant, conserved, and prevalent bacteriophages in the human gut is crAssphage [46], a finding that suggests some phages may be highly conserved in the human population.

The impact of bacteriophages on human health is under active consideration. Phage therapy entails the isolation and inoculation of phages (or their antibacterial enzymes) that target a specific bacterium. While not all phage treatments are effective [47], several in vitro and in vivo experiments have been successful. As a treatment for CDI, 10^8^ plaque-forming units per mL of a specific phage were introduced into a human colon model. Over a period of 35 days, the treatment caused a significant decrease in vegetative *C*. *difficile* cells (albeit there was an increase in *C*. *difficile* spores) as well as toxin production to levels below the detection threshold of the assay [48]. Control replicates contained high concentrations of both vegetative cells and toxin. Phage therapy of CDI in a hamster model also significantly delayed bacterial colonization and the onset of symptoms [49]. Specific phage cocktails could, in theory, allow commensal bacteria that are in competition with *C*. *difficile* to reflourish in the gut [50]. While *C*. *difficile* phages may eventually be developed into therapeutic agents, there is yet no evidence that phages specific to *C*. *difficile* are transferred in fecal transplants.

There have been several concerns about the safety of phage therapy. To alleviate the apprehension, a recent human clinical trial orally inoculated a group of 15 subjects with a high dose of 17 phage groups targeting *Escherichia coli* and *Proteus* infection and found no adverse effects [51]. Phage therapy cocktails have continuously demonstrated potential to target and eliminate specific virulent bacteria while avoiding adverse effects typical of antibiotics (e.g., resistance, diarrhea, etc.) [52–54]. However, a potential drawback is the risk of evolution of bacterial resistance to phages [55,56], though phages can potentially evolve counter-resistance mechanisms. Furthermore, human studies involving phage therapy are relatively small-scale thus far. Larger patient cohorts and further studies of phage dosages, evolution of phage host ranges and bacterial resistance [56], and the stability of phage-based drugs are needed.

### Archaea and Fungi

Archaea are well-recognized but relatively understudied members of the human gut microbiota [57], with methanoarchaeon comprising up to 10% of fecal anaerobes [20]. Based on the concentration of methane in breath, estimates suggest a minimum presence of 10^7^–10^8^ methanogens per gram of both dry and wet stool [58,59], though it is unresolved what percentage of these methanogens are from archaea versus bacteria. Higher than normal concentrations of intestinal archaea are associated with Crohn’s disease and multiple sclerosis [60]. Similarly, fungi in the gut have been cultured in 70% of healthy adults [61]. They occur in estimated concentrations of up to 10^6^ microorganisms per gram of feces [62] and appear to comprise only 0.03% of all microbes in feces [63]. *Candida albicans* is the most common and studied yeast, but it is kept in check by competitive commensal bacteria in a healthy gut. When bacterial homeostasis is disturbed, however, *C*. *albicans* increases its numbers drastically [64,65]. These fungi may also help induce intestinal diseases by penetrating the intestinal colonocyte barrier and driving inflammation [66]. Indeed, high concentrations of *C*. *albicans* occur in individuals with inflammatory bowel diseases [67,68]. The contribution of archaea and fungi to changes in function will be an important area of future research.

### Human Colonocytes

Interestingly, viable epithelial cells of the large intestine, or colonocytes, can be isolated at a concentration of up to 10^7^ per gram of wet fecal material [23]. Viable colonic cells have effectively been isolated from newborn fecal samples (>80% viable) [21] and biopsy specimens from colonic crypts (>98% viable) [22]. Isolation is possible due to the resilient ability of colonocytes to take on a globular shape and survive once exfoliated into the fecal stream [69]. Thus, their viability and partial functionality is likely retained in the course of some transplant treatments, especially in animal models that utilize feeding or oral gavage of fecal material.

By acting as the physical barrier between bacteria and the host’s internal tissues and organs, colonocytes allow host tolerance of the intestinal microbiota [70]. When high levels of colonocyte death occur, their mediating role disintegrates because of increased intestinal permeability [71]. Indeed, major pathological conditions of the bowel are associated with changes in the growth and functions of the colonic epithelium [22,72], similar to changes frequently observed in microbiota studies. Their restoration is key in successful recovery from such conditions. A recent study transplanted healthy viable colon stem cells into an immunodeficient mouse model with superficial colon damage and found that cells readily integrated, and a single layer of epithelium fully covered areas lacking colonocytes [73]. The presence of colonic stem cells in feces has yet to be recorded, although one study recovered stem cells from the colonic epithelium that often sheds into the fecal stream [74]. Should colonic stem cells be identified in feces in human or animal models, they may affect the success of transplants if they can engraft in recipients.

In addition to colonocytes, molecules such as immunoglobulin A (IgA) can act as the first line of defense for the intestinal epithelium [75]. IgA reinforces the intestinal barrier and protects host cells against pathogens and enteric toxins in the gut [75]. For instance, IgA significantly inhibited *C*. *difficile* toxin binding to hamster intestinal brush border membranes compared to the control [76]. Likewise, human epithelial cell lines with IgA added to their surface showed a decrease in *C*. *difficile*–associated pathology compared to cells lacking IgA [77]. It remains to be seen if introducing IgA directly into human subjects will be beneficial.

### Metabolites

It is well known that fiber is metabolized by intestinal bacteria to produce short-chain fatty acids (SCFA) that have prominent anti-inflammatory and T cell–inducing properties in the colon [78–80]. Fiber strongly contributes to fecal weight, and low fiber diets in mice can lead to an irreversible loss in bacterial diversity [81]. While direct reintroduction of missing fiber in this study did not restore the diversity, transplants from mice with a high fiber diet did. Furthermore, low fiber diets lead to “microbial starving,” whereby once-commensal bacteria attack the intestinal lining [82]. Fiber supplements used in a study with *C*. *difficile*–infected hamsters, however, managed to significantly modulate onset time of systemic symptoms [83]. Fiber intake has also been linked to increased microbial diversity and reduced obesity in humans [84,85].

Butyrate-producing bacteria or butyrate concentrations in feces can be lower in patients with colorectal cancer and ulcerative colitis [86–88]. Preliminary studies of enemas with butyrate or SCFA cocktails (acetate, butyrate, and propionate) show some resolution in patients with distal ulcerative colitis [89–93]. Following these treatments, 35%–67% of patients exhibited improvement. Furthermore, oral administration of sodium butyrate in a colitis mouse model alleviated inflammation and mucosal damage [94], and propionate led to improvement of symptoms in a multiple sclerosis mouse model by promoting regulatory T cell differentiation [95]. No adverse side effects were noted in any of these studies, though some metabolite enemas are malodorous. One review, however, cautions against the use of such metabolites [96]. While butyrate acts as an energy source, increases colonocyte growth, and decreases apoptosis of colonocytes under healthy conditions [97], excess butyrate accumulation around human colonic carcinoma cells has been connected with increased apoptosis [98]. Finally, estimates suggest there are nearly 900 gene clusters in human gut–associated bacteria that make small molecules [99]. Determining functions may be important in understanding the composite nature of feces and its effects on fecal transplants in humans and/or animal models.

## Summary

Here, we cautiously note that bacteria, either viable or unviable in transferred fecal material, may not be the only player in donor feces that affects the recipient's biology. On the one hand, the effects of bacteria on CDI or animal model traits such as obesity [100] and toxin tolerance [101] appear well justified thus far. On the other hand, in a broader context where fecal transplants are solely utilized in animal model studies and other human diseases, judicious reductionism seems warranted in light of a limited understanding of the complex nature of feces. Deconstructing the benefit and interactions of constituent parts of fecal matter will clarify the relative importance and causality of each of these components and the potential development of specific therapies.

**Key Points and Future Directions**

- A few studies using cocktails of bacteria in animal models and humans show suppression of CDI. However, these studies are preliminary and limited.
- Through bacterial targeting, phage therapy can potentially eliminate virulent bacteria in a diseased gut and allow commensal bacterial to reflourish.
- Colonocytes prevent bacterial translocation into internal tissues and organs; transplants of healthy viable colon stem cells into mouse models result in repair of superficial colon damage.
- Metabolites can nourish the colonocyte barrier and intestinal bacteria. Oral administration of metabolites can alleviate inflammation, mucosal damage, and multiple sclerosis symptoms. However, only 5% of the SCFAs produced in the distal colon are estimated to be excreted into feces [102]. Hence, metabolite concentrations are likely to be much lower than concentrations used in oral administration studies.
- Archaea and fungi are common in feces. Though high concentrations of intestinal archaea and certain fungi have been correlated to both intestinal and autoimmune diseases, their causative effects are unknown.
- Human genetic relatedness has little to no influence on the effectiveness of human fecal transplants, though genetic factors do shape bacterial community composition.
- Individual components of fecal matter can yield health benefits and may work synergistically to restore homeostasis. There is a cautious need for continued reductionism to understand the precise benefit and interactions of various fecal components.

## Abbreviations

CDI: *Clostridium difficile* infection
IgA: immunoglobulin A
SCFA: short-chain fatty acids
